# Reviewed article: Silva MT, Amado DM, Rocha PRS, Barreto JO. Integrative and complementary healthcare practices for hypertension: a summary of recommended clinical guidelines. Epidemiol Serv Saude. 2025:34;e20240844

**DOI:** 10.1590/S2237-96222025v34e20240844.a

**Published:** 2025-08-04

**Authors:** Pedro Henrique Brito da Silva

**Affiliations:** Secretaria Municipal de Saúde de Senador Canedo

**Table:** 

Rating	Excellent	Good	Average	Below average	Poor
Interest	✓				
Quality			✓		
Originality			✓		
Overall			✓		

**Table:** 

Questionnaire	Yes	No	Not applicable
Does the manuscript contain new and significant information to justify publication?	✓		
Does the Abstract (Summary) clearly and accurately describe the content of the article?		✓	
Is the problem significant and concisely stated?	✓		
Are the methods described comprehensively?	✓		
Are the interpretations and conclusions justified by the results?		✓	
Is adequate reference made to other work in the field?	✓		

## Manuscript Structure

Length of article is: Adequate

Number of tables is: Adequate

Number of figures is: Adequate

Please state any conflict(s) of interest that you have in relation to the review of this paper (state “none” if this is not applicable): None

## Comments

O artigo apresenta dados interessantes sobre o uso das Práticas Integrativas e Complementares (PICS) no manejo da hipertensão arterial, mas necessita de uma revisão mais ampla em vários aspectos. Embora a síntese de evidências seja relevante, a exclusão de diretrizes que utilizam outros sistemas de avaliação além do GRADE limita a abrangência dos resultados. Além disso, faltam dados quantitativos mais detalhados, o que compromete a robustez das conclusões. A seção de discussão poderia ser mais crítica, explorando com mais profundidade as limitações metodológicas dos estudos incluídos, como o viés de seleção e a qualidade das amostras.

A relação entre o objetivo do estudo, a hipótese de pesquisa e os resultados não está suficientemente clara, o que dificulta a compreensão do foco central da pesquisa. Recomenda-se uma revisão que amplie o escopo da análise, forneça mais detalhes quantitativos e proponha recomendações mais claras para futuras investigações. Com essas melhorias, o artigo terá um maior impacto e uma contribuição mais sólida para a literatura científica. As sugestões estão em anexo. 

